# STRENGTHENING PERSONAL AND COMMUNITY DISASTER SUPPORT NETWORKS OF OLDER ADULTS

**DOI:** 10.1093/geroni/igz038.2166

**Published:** 2019-11-08

**Authors:** Sato Ashida, Lena Thompson, Erin L Robinson

**Affiliations:** 1 College of Public Health, The University of Iowa, Iowa City, United States; 2 University of Iowa College of Public Health, Iowa City, Iowa, United States; 3 School of Social Work, Univesrsity of Missouri, Columbia, Missouri, United States

## Abstract

The Ecological Model posits a multilevel approach to understanding of and strengthening social networks of community based older adults. We present findings from our intervention, network analysis, and efforts at influencing policy making at the state level to better prepare them for pre and post disaster and emergency situations. At the individual-level, we implemented the Disaster PrepWise program to help community-based older adults develop personal disaster plans. We found increases in personal emergency network size by an average of three non-familial individuals. At the community-level, we evaluated two disaster management networks in Eastern Iowa counties. We found strong collaborations in disaster planning and response among 44 governmental and community-based organizations, but weaker collaborations in supporting older residents, suggesting a need in this area. At the policy-level, we are developing a state-level network of organizations to address policy barriers to effectively support older Iowans.

